# Chimeric autoantibody receptor T cells for targeted depletion of myeloperoxidase-specific anti-neutrophil cytoplasmic antibody-producing B cells

**DOI:** 10.3389/fimmu.2026.1814452

**Published:** 2026-05-08

**Authors:** Heather M. Sosnoski, William Aguilar, Shawna K. Brookens, David A. Degaramo, John T. Keane, Hyeon-Gyu S. Lewis, Damodar Gullipalli, Wenchao Song, Avery D. Posey

**Affiliations:** 1 Systems Pharmacology and Translational Therapeutics, University of Pennsylvania Perelman School of Medicine, Philadelphia, PA, United States; 2 Corporal Michael J. Crescenz VA Medical Center, Philadelphia, PA, United States

**Keywords:** ANCA vasculitides, autoimmune diseases, B cell depletion therapies, chimeric autoantibody receptor (CAAR), targeted therapy

## Abstract

Anti-neutrophil cytoplasmic antibody (ANCA) vasculitides are autoimmune disorders driven by autoantibodies against neutrophil granule proteins, myeloperoxidase (MPO) and proteinase 3, which cause necrotizing small-vessel inflammation with prominent lung and kidney involvement, resulting in pulmonary hemorrhage and glomerulonephritis. Current remission-induction regimens rely on broad immunosuppression and global B cell depletion, which are effective but associated with substantial infection risk and relapse that often necessitates repetitive, long-term treatment. Here, we develop MPO chimeric autoantibody receptor (CAAR) T cells as a target-specific therapy to eliminate MPO-autoreactive B cells. MPO CAAR T cells selectively eliminated anti-MPO BCR-expressing B cell lines *in vitro* and *in vivo*. In primary murine autoreactive B cell cultures, MPO CAAR T cells suppressed anti-MPO IgG production without inducing global B cell depletion. Together, these findings support MPO CAAR T cells as a precision approach to eliminate pathogenic B cells in ANCA vasculitis, potentially eliminating the need for generalized humoral immunosuppression.

## Introduction

Anti-neutrophil cytoplasmic antibody (ANCA) vasculitides comprise a group of uncommon, age-associated autoimmune diseases characterized by necrotizing inflammation of small blood vessels, most prominently affecting the lungs and kidneys and leading to pulmonary hemorrhage and glomerulonephritis (1–3). Historically classified as granulomatosis with polyangiitis (GPA) or microscopic polyangiitis, the defining pathologic feature of ANCA vasculitides is the presence of autoantibodies directed against the neutrophil granule proteins myeloperoxidase (MPO) or proteinase 3 (PR3) (1, 4, 5). During inflammatory conditions, neutrophils externalize MPO and PR3, rendering these antigens accessible to circulating anti-MPO B cells, triggering B cell expansion and production of ANCA (6–9). ANCA engagement amplifies neutrophil activation, driving oxidative burst and degranulation, altering adhesion dynamics at the vascular wall, recruiting and activating T cells, triggering the alternative complement pathway, and ultimately promoting endothelial injury and vascular destruction (10, 11). Beyond antibody secretion, pathogenic B cells also function as antigen-presenting cells (APCs), contributing to disease by sustaining autoreactive T cell responses (12, 13). Consistent with these central roles, B cell depletion has become an effective strategy for inducing remission in the majority of ANCA patients (14).

Without treatment, approximately 20% of patients with ANCA vasculitis die within the first year, with mortality increasing to 47% after progression to end-stage renal disease (1). Current remission-induction regimens, most commonly cyclophosphamide and/or rituximab (anti-CD20), achieve remission in the majority of patients and have improved long-term outcomes (15, 16). Despite these improvements, ANCA vasculitis remains associated with substantial mortality, and treatment-related toxicity has emerged as a dominant driver of morbidity and mortality, particularly in the first year of therapy (15, 17). Severe infections occur in nearly one-quarter of patients, are most common early after treatment initiation, and remain a persistent risk for the duration of immunosuppressive therapy (18). Moreover, frequent relapse after B cell repopulation necessitates repeated or long-term treatment, prolonging these risks (19, 20). Consequently, infection and sepsis attributable to immunosuppression now account for more deaths than vasculitis itself (1, 21). These limitations underscore the need for durable, targeted therapies that selectively eliminate autoreactive B cells while preserving protective humoral immunity.

In recent years, anti-CD19 CAR T cell therapy has been applied to autoreactive B cell–driven autoimmune diseases, providing deeper and more durable B cell depletion than monoclonal antibody therapies (22–24). In ANCA vasculitis, anti-CD19 CAR T cells have shown promising results in murine studies and early clinical case reports, although experience in MPO-ANCA vasculitis patients remains limited to date (22, 25, 26). In this study, we generate chimeric autoantibody receptor (CAAR) T cells as a targeted therapy for MPO-ANCA vasculitis without global inhibition of humoral immunity. We found that murine MPO (mMPO) CAAR T cells are specifically activated by and selectively eliminate B cell lines engineered to express anti-MPO B cell receptors (BCRs) *in vitro*. In a murine anti-mMPO BCR-expressing B cell tumor model, mMPO CAAR T cells reduce tumor burden *in vivo* without overt toxicity. Finally, using primary murine autoreactive B cells, we demonstrate that mMPO CAAR T cells suppress anti-MPO IgG production without inducing global B cell depletion. Together, these findings support mMPO CAAR T cells as a precision strategy to eliminate MPO-reactive B cells while avoiding the broad immunosuppression that limits current therapies for MPO-ANCA vasculitis.

## Results

### Engineering and validation of murine MPO CAAR T cells

**Figure 1:**
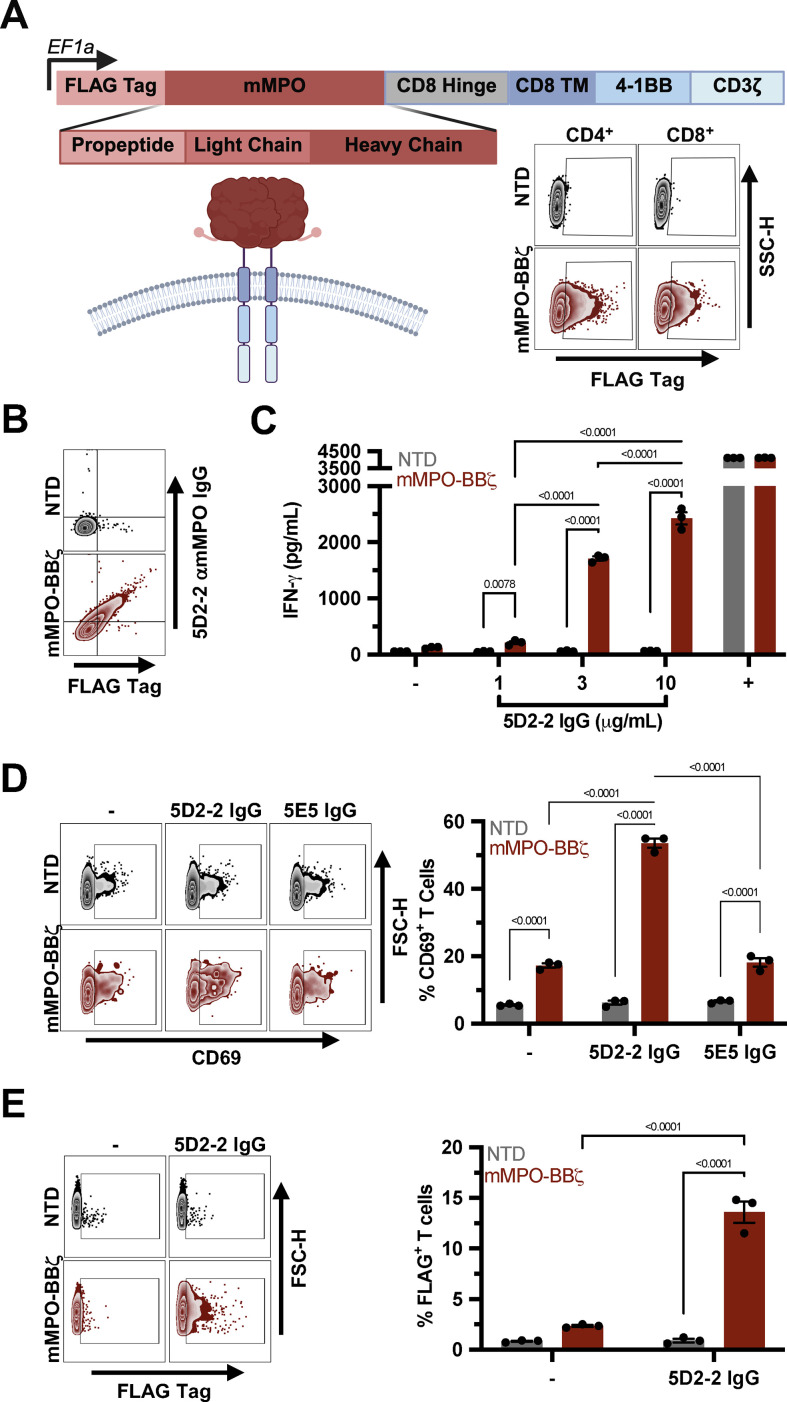
mMPO-BBζ T cells bind anti-mMPO IgG and undergo antigen-dependent activation. **(A)** Schematic of murine MPO CAAR (mMPO-BBζ) comprising extracellular murine MPO with an N-terminal FLAG tag, human CD8α hinge/transmembrane domain, 4-1BB costimulatory domain, and CD3ζ signaling domain. Representative flow cytometry of mMPO-BBζ surface expression on transduced primary human CD4^+^ and CD8^+^ T cells, assessed by anti-FLAG staining. **(B)** Binding of 5D2–2 anti-mMPO IgG to NTD versus mMPO-BBζ T cells assessed by anti-murine Ig and anti-FLAG staining. **(C)** IFN-γ secretion by NTD and mMPO-BBζ T cells in supernatants 48 h after stimulation with increasing concentrations of plate-bound 5D2–2 IgG, measured by ELISA; unstimulated (−) and PMA/ionomycin (+) conditions served as negative and positive controls respectively. **(D)** CD69 expression after 48h stimulation with 10 μg/ml plate-bound 5D2–2 IgG or irrelevant IgG1 antibody 5E5 shown as representative flow cytometry (*left*) and quantified percent of T cells (*right*). **(E)** mMPO CAAR expression, determined by FLAG staining, 5D2–2 IgG stimulation (*left*) and quantified as %FLAG^+^ (*right*). Data are shown as mean ± SEM. Statistical significance for **(C)** was assessed by two-way ANOVA with Sidak’s multiple comparison test, and **(D, E)** by two-way ANOVA with uncorrected Fisher’s LSD.

To enable targeted depletion of MPO-autoreactive B cells, we engineered a murine MPO CAAR (mMPO-BBζ) comprising extracellular mMPO (pro-peptide, light chain, and heavy chain) with an N-terminal FLAG tag and C-terminal human CD8α hinge/transmembrane domain and 4-1BB/CD3ζ signaling domains (**Figure 1A**). To generate MPO CAAR T cells, primary human CD4^+^ and CD8^+^ T cells were activated with anti-CD3/CD28, transduced with mMPO-BBζ lentivirus, and expanded in interleukin 2 (IL-2). Surface CAAR expression was confirmed by anti-FLAG staining, with detectable expression in both CD4^+^ and CD8^+^ subsets (**Figure 1A**).

CAAR activity requires recognition of anti-mMPO immunoglobulin, so we tested binding and activation using the anti-mMPO IgG monoclonal antibody, 5D2-2. 5D2–2 IgG selectively bound mMPO-BBζ+ T cells (anti-mouse Ig staining) (**Figure 1B**). Plate-bound 5D2–2 IgG induced T cell activation, including increased CD69 expression and IFN-γ secretion as well as antigen-dependent CAAR enrichment (**Figures 1C–E**). mMPO-BBζ T cells were not activated by an irrelevant IgG1 antibody (5E5). Together, these data demonstrate that mMPO-BBζ can be expressed on primary human T cells, bind to anti-MPO IgG, and mediate antigen-dependent activation.

### mMPO CAAR T cells selectively lyse anti-mMPO–expressing target cells *in vitro*

To model CAAR target recognition, we cloned the variable regions of both 5D2–2 IgG and a second anti-mMPO hybridoma line (6G1-1) and generated a 5D2–2 and 6G1–1 single-chain variable fragment (scFv)–based 4-1BB/CD3ζ chimeric antigen receptors (CAR) for validation studies (**Supplementary Figures 1A–S1E**). 5D2-2-BBζ and 6G1-1-BBζ T cells preferentially lysed mMPO-expressing HEK293T cells compared with WT HEK293T controls, confirming the expected antigen specificity of the 5D2–2 and 6G1-1-derived binding domains (**Supplementary Figures 1F–S1H**).

**Figure 2:**
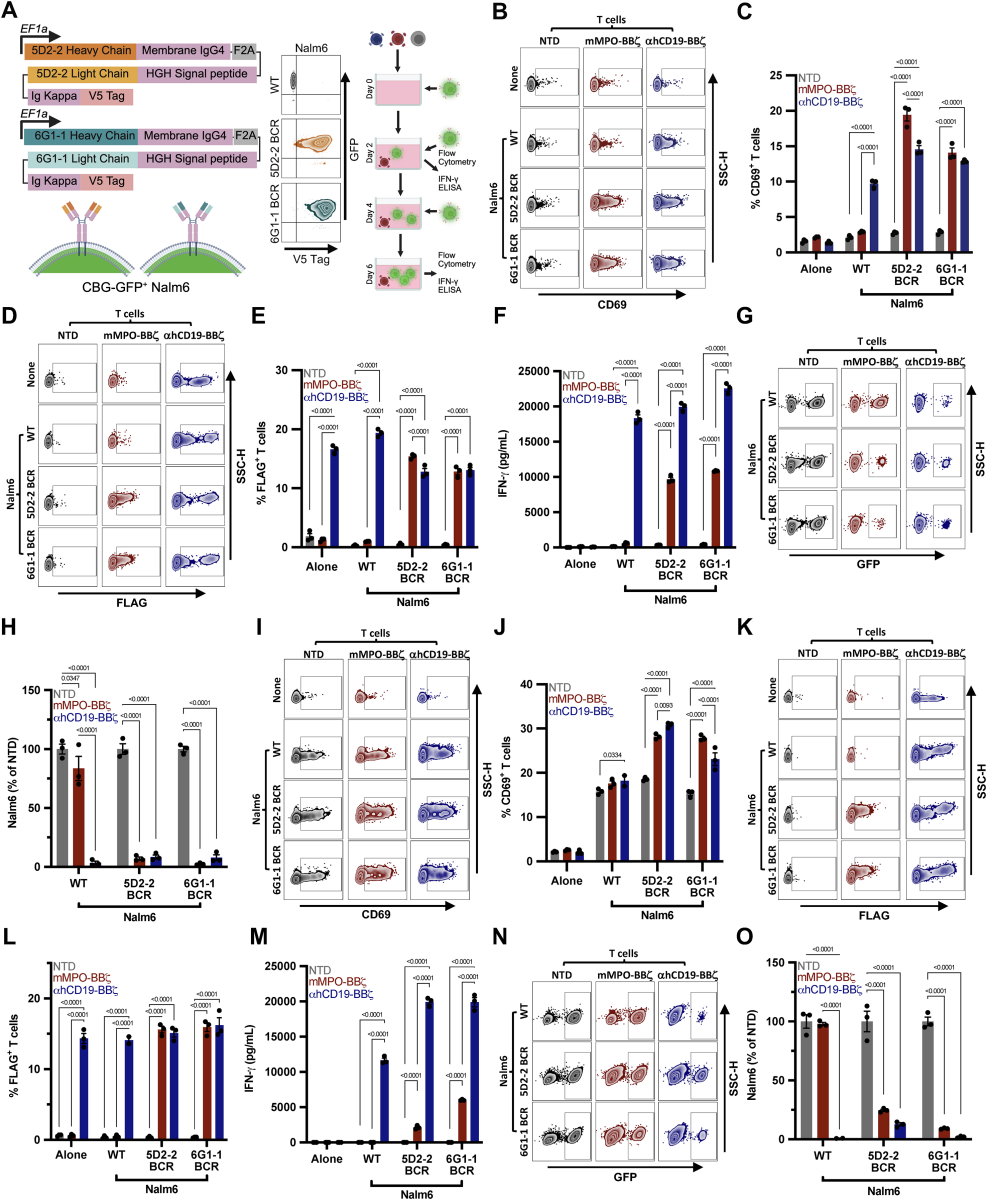
mMPO-BBζ T cells selectively deplete 5D2–2 BCR-expressing Nalm6 targets *in vitro.* **(A)** Schematic of the 5D2-2 and 6G1-1 BCR expression construct (*left*) consisting of 5D2–2 or 6G1–1 variable heavy chain, membrane IgG4, F2A self-cleaving peptide, human growth hormone (HGH) signal peptide, 5D2–2 or 6G1–1 variable light chain, Ig Kappa, and a V5 tag. Expression of 5D2-2 or 6G1-1 BCR (V5 tag) and GFP in Nalm6 (*middle*). Schematic of repeated stimulation of NTD, mMPO-BBζ, and αhCD19-BBζ T cells over six days with Nalm6 targets added to co-culture every two days (*right*). **(B)** Representative flow cytometry of CD69 upregulation by NTD, mMPO-BBζ, and αhCD19-BBζ T cells alone and after co-culture with WT, 5D2–2 BCR, or 6G1–1 BCR Nalm6 at 1:1 E:T ratio. **(C)** Quantification of CD69 expression in NTD and mMPO-BBζ T cells alone and after co-culture with WT, 5D2–2 BCR, or 6G1–1 BCR Nalm6, displayed as %CD69^+^ cells. **(D)** Representative flow cytometry of CAAR expression (FLAG tag) by NTD and mMPO-BBζ, and αhCD19-BBζ T cells alone and after co-culture with WT, 5D2–2 BCR, or 6G1–1 BCR Nalm6 at a 1:1 E:T ratio. **(E)** Quantification of CAAR expression in NTD and mMPO-BBζ T cells alone and after co-culture with WT and 5D2–2 BCR Nalm6, displayed as %FLAG^+^ cells. **(F)** IFN-γ secretion after 48 h co-culture of NTD, mMPO-BBζ, and αhCD19-BBζ T cells alone and with WT, 5D2–2 BCR, or 6G1–1 BCR Nalm6 at 1:1 E:T. **(G)** Representative flow cytometry of live GFP^+^ Nalm6 **(H)** quantified as fold change relative to NTD T cells. **(I)** Representative flow cytometry plot and **(J)** quantification of CD69^+^ T cells after repeated stimulation with WT versus 5D2–2 BCR or 6G1–1 BCR Nalm6. **(K)** Representative flow cytometry plot and **(L)** quantification of CAAR surface expression on mMPO-BBζ T cells across repeat stimulations. **(M)** IFN-γ secretion by NTD, mMPO-BBζ, and αhCD19-BBζ T cells after three consecutive 48h co-cultures with WT versus 5D2–2 BCR or 6G1–1 BCR Nalm6. **(N)** Representative flow cytometry plots from the repeat-stimulation assay showing viable Nalm6 cells **(O)** quantified as fold change relative to NTD T cells. Data are shown as mean ± SEM. Statistical significance for panels **(C, E, F, H, J, L, M, O)** was assessed by two-way ANOVA with Tukey’s multiple comparisons test **(C, F, H, J, M, O)** or uncorrected Fisher’s LSD **(E, L)**.

We next engineered B-lineage cells, Nalm6, to express a membrane-tethered 5D2–2 or 6G1–1 scFv (5D2-2-TM and 6G1-1-TM). Both 5D2-2-TM and 6G1–1 Nalm6 induced IFN-γ production by mMPO-BBζ T cells exclusively and were preferentially depleted relative to WT Nalm6 (**Supplementary Figure 2A–S2E**). To more closely mimic autoreactive B cells, we generated Nalm6 cells expressing a full 5D2–2 or 6G1–1 B cell receptor (BCR) (**Figure 2A**). In co-culture, mMPO-BBζ T cells upregulated CD69 upon engagement with both 5D2–2 BCR and 6G1–1 BCR Nalm6 but not WT Nalm6, whereas αhCD19-BBζ T cells upregulated CD69 in response to all targets (**Figures 2B, C**; **Supplementary Figures 3B, C**). Upon activation, both mMPO-BBζ^+^ and αhCD19-BBζ^+^ T cells expanded and secreted IFN-γ (**Figures 2D–F**; **Supplementary Figures 3D–S3F**). Consistent with activation, mMPO-BBζ T cells preferentially depleted 5D2–2 BCR and 6G1–1 BCR Nalm6 while sparing WT Nalm6, determined by live, GFP-expressing cells detected by flow cytometry. As expected αhCD19-BBζ T cells depleted all Nalm6 targets (**Figures 2G, H**; **Supplementary Figure 3G, S3H**).

To assess durability, we performed repeated antigen challenges by adding fresh Nalm6 targets to co-cultures and replacing half of the culture medium every 48h. After three stimulations, mMPO-BBζ T cells maintained 5D2–2 BCR and 6G1–1 BCR target-selective CD69 upregulation, CAAR expression, and IFN-γ secretion and continued to deplete 5D2–2 BCR and 6G1–1 BCR Nalm6 relative to WT Nalm6 (**Figures 2I–O**; **Supplementary Figure 3I–S3O**). These results support selective and persistent effector function of mMPO-BBζ CAAR T cells against anti-mMPO BCR-expressing targets *in vitro*.

### mMPO CAAR T cells mediate *in-vivo* control of anti-mMPO BCR-expressing tumors

**Figure 3:**
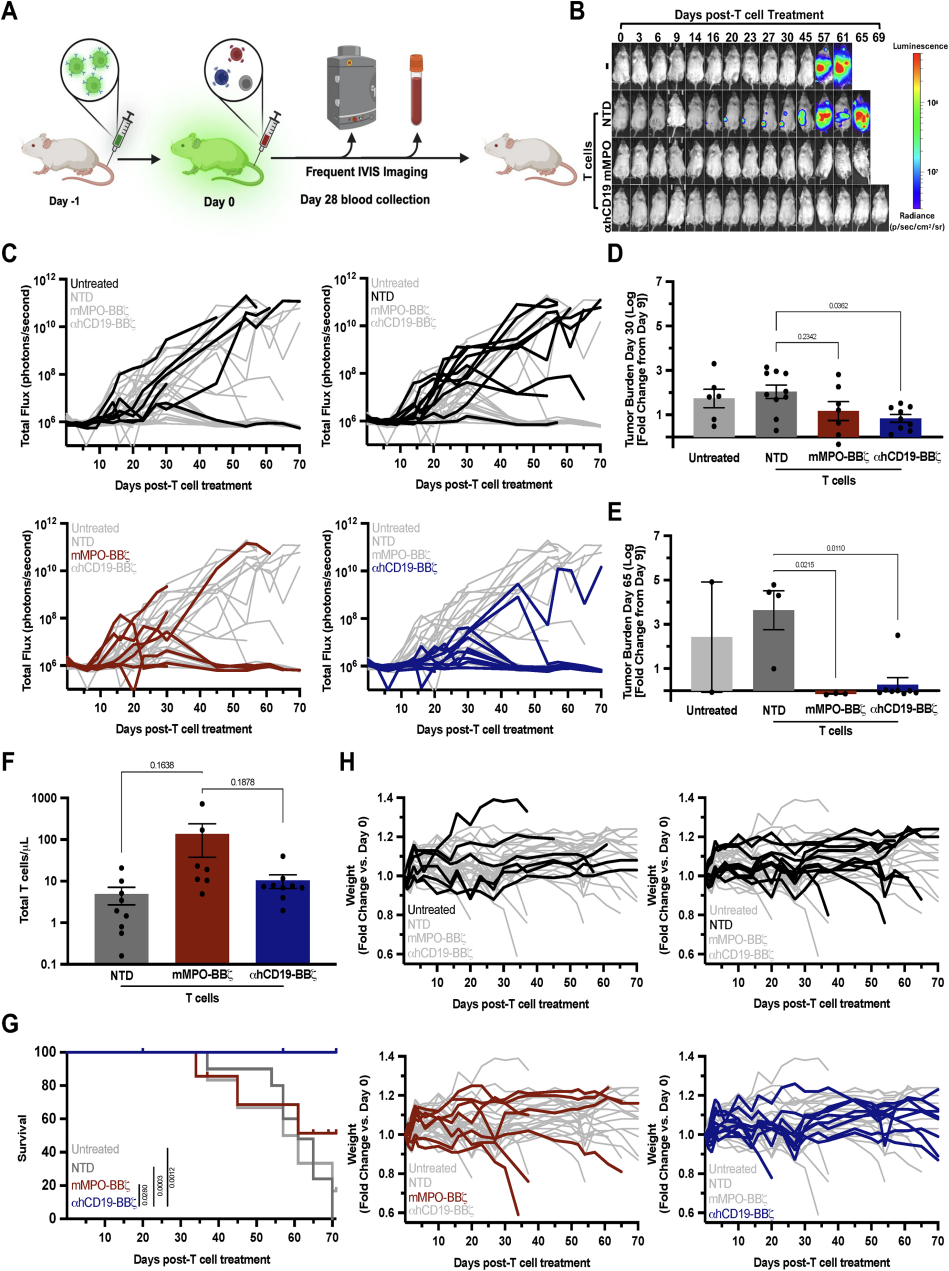
mMPO-BBζ T cells control anti-mMPO B cells *in vivo* and persist in peripheral blood. **(A)** Experimental schema for NSG engraftment with 1 × 10^6^ 5D2–2 BCR Nalm6 on day -1 followed by treatment with 2 × 10^6^ mMPO-BBζ^+^ (*n* = 7), or αhCD19-BBζ^+^ (*n* = 10) T cells on day 0, serial bioluminescence imaging, and peripheral blood collection for analysis. Untreated mice (*n* = 6) and treatment with NTD T cells equal to the total number of mMPO-BBζ T cells (*n* = 10) served as a negative control. **(B)** Representative serial bioluminescence images of 5D2–2 BCR Nalm6 tumor burden. **(C)** Bioluminescent imaging [BLI; total flux (photons/second)] quantification over time for each treatment group plotted longitudinally: untreated (top left), NTD (top right), mMPO-BBζ (bottom left) or αhCD19-BBζ (bottom right). **(D)** Tumor burden on day 30 graphed as fold change from tumor burden on day 9. **(E)** Tumor burden on day 65 graphed as fold change from tumor burden on day 9. **(F)** Peripheral blood human T cell persistence on day 28 post-treatment quantified by flow cytometry as human CD3^+^ cells/μl blood. **(G)** Kaplan-Meier survival curve by treatment group. **(H)** Mouse body weight over time by treatment group graphed as fold change from day 0. Data in panels **(D–F)** are shown as mean ± SEM. Statistical significance of **(D–F)** was assessed by ordinary one-way ANOVA with Tukey’s multiple comparisons test. Survival **(G)** was analyzed by Kaplan-Meier with log-rank (Mantel-Cox) testing.

To test the *in-vivo* activity of CAAR T cells, Nod *scid* gamma (NSG) mice were engrafted with 5D2–2 BCR Nalm6 and treated with mMPO-BBζ T cells the next day. Untreated and NTD T cell–treated mice served as negative controls, and αhCD19-BBζ groups served as positive controls (**Figure 3A**). Serial bioluminescent imaging demonstrated progressive tumor growth in untreated and NTD-treated mice, whereas mMPO-BBζ treatment produced tumor control in a subset of animals and αhCD19-BBζ produced near uniform tumor eradication (**Figures 3B–E**; **Supplementary Figure 4A**). Human T cells were detectable in peripheral blood at day 28 across treated groups, consistent with *in-vivo* persistence (**Figure 3F**; **Supplementary Figures 4B–S4E**). Across the study, mMPO-BBζ treatment did not cause weight loss or fatality beyond that observed in control T cell groups, supporting treatment tolerability in this model (**Figures 3G, H**). Collectively, these data demonstrate the *in-vivo* anti-tumor activity of mMPO CAAR T cells against anti-MPO BCR-expressing targets.

### mMPO CAAR T cells selectively deplete primary anti-MPO B cell responses *ex vivo*

**Figure 4:**
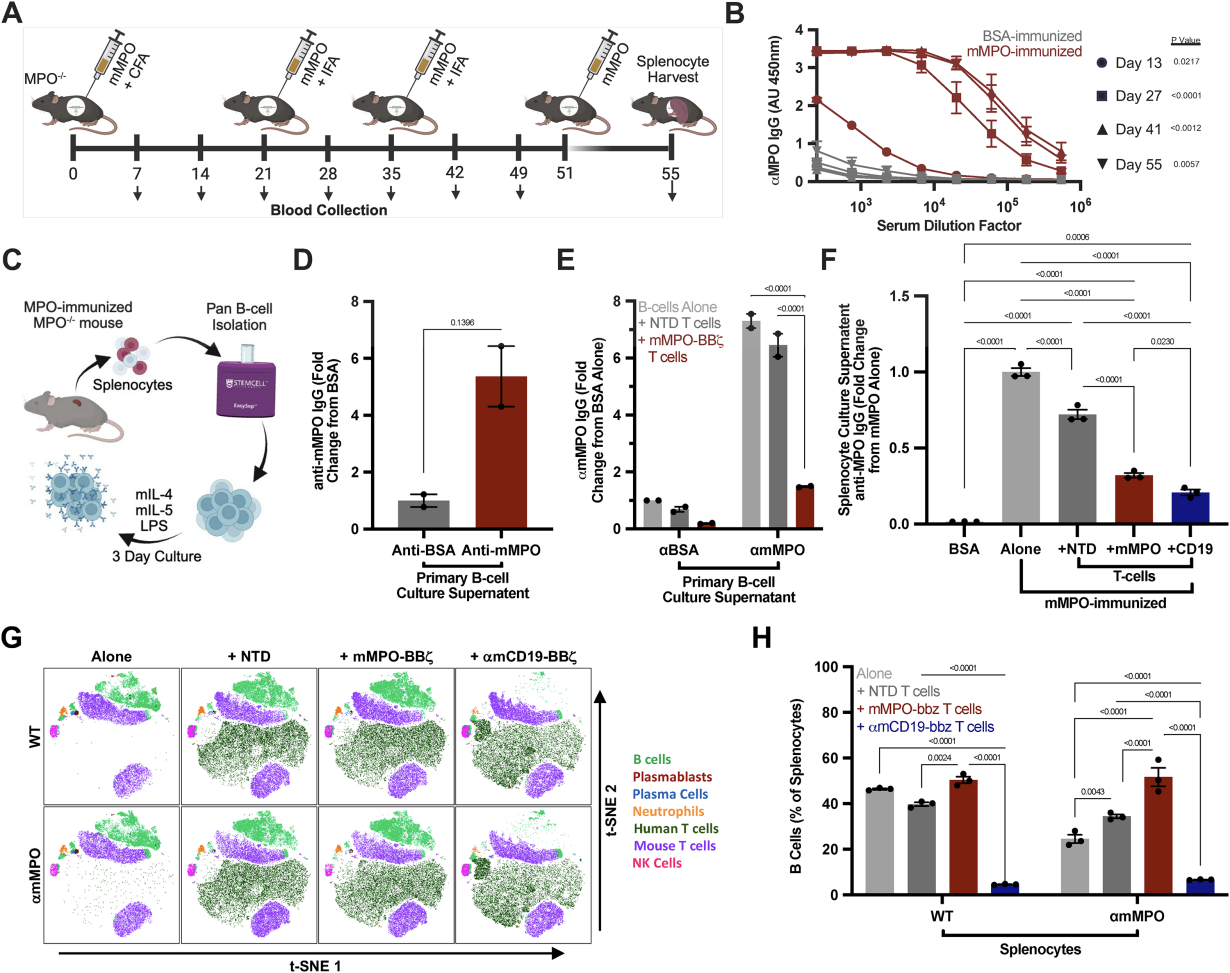
mMPO-BBζ T cells selectively deplete primary anti-mMPO B-cell responses *ex vivo*. **(A)** Immunization schema for generating anti-mMPO responses in MPO^−/−^ mice. Mice were injected intraperitoneally with 10 μg BSA or mMPO on day 0 in complete Freund’s adjuvant (CFA) followed by injections of 10 μg BSA or mMPO in incomplete Freund’s adjuvant (IFA) on days 21 and 35. Finally, mice received a booster of 10μg BSA or mMPO in PBS three days prior to splenocyte harvest. Peripheral blood was collected bi-weekly to determine serum anti-mMPO IgG titers. **(B)** Bi-weekly serum anti-mMPO IgG titers over time quantified by ELISA and graphed as absorbance units (AU; 450 nm–540 nm) over a range of 250X-546750X serum dilutions. **(C)** Workflow for isolating B cells or splenocytes and culturing under plasma cell-differentiating conditions (mIL-4, mIL-5, LPS). Anti-mMPO IgG in supernatants from B cells derived from BSA- versus MPO-immunized donors. **(E)** Anti-mMPO IgG after co-culture of purified B cells with NTD or mMPO-BBζ T cells for three days *ex vivo*, quantified by ELISA and graphed as the fold change from anti-BSA B-cells alone. **(F)** Anti-mMPO IgG after co-culture of pooled bulk splenocytes with NTD, mMPO-BBζ, or αmCD19-BBζ T cells for three days, quantified by ELISA and graphed as the fold change from mMPO-immunized *MPO^-/-^* splenocytes cultured alone. **(G)** t-SNE visualization of immune subsets (B cells, plasma blasts, plasma cells, neutrophils, human T cells, murine T cells, and NK cells) after 3 days of culture of bulk BSA- or mMPO-immunized splenocytes alone or with NTD, mMPO-BBζ, or αmCD19-BBζ T cells. **(H)** Quantification of B cells (including plasma blasts, and plasma cells) from **(G)** graphed as percent of total splenocytes in culture. Data in panels **(B, D–F)** are shown as mean ± SEM. Statistical significance for B was assessed by ordinary one-way ANOVAs between week-matched BSA- and mMPO-immunized mice. Statistical significance for **(D)** was assessed by unpaired T test with Welch’s correction. Statistical significance for **(E)** was assessed by two-way ANOVA with Tukey’s multiple comparisons test. Statistical significance for **(F)** was assessed by one-way ANOVA with Tukey’s multiple comparisons test.

To evaluate primary autoreactive responses, we produced and purified murine MPO and immunized MPO^−/−^ mice to generate anti-MPO B cell responses (**Figures 4A, B**; **Supplementary Figures 5A–S5E**). Immunized mice developed serum anti-mMPO IgG titers over time (**Figure 4B**). B cells isolated from immunized mice secreted anti-MPO IgG under plasma cell-differentiating conditions (**Figures 4C, D**). Co-culture of these B cells with mMPO-BBζ T cells reduced anti-MPO IgG in supernatants compared with untreated and NTD T cell-treated controls, consistent with depletion of anti-MPO antibody-producing cells (**Figure 4E**; **Supplementary Figure 5G**). Co-culture with bulk splenocytes yielded the same results (**Figure 4F**). In phenotyping assays, αmCD19-BBζ T cells induced near-complete depletion of B cells from splenocyte culture after 3 days, whereas mMPO-BBζ T cells preserved non-target immune subsets, supporting antigen-selective activity (**Figures 4G, H**; **Supplementary Figure 5G**).

### *In-vivo* generated anti-mMPO immunoglobulin binds mMPO CAAR T cells without substantially impairing target killing *in vitro*

A potential limitation of CAAR T cells is competitive inhibition by circulating autoantibodies. Plate-bound 5D2–2 IgG triggered dose-dependent IFN-γ secretion by mMPO-BBζ T cells, whereas soluble 5D2–2 IgG did not measurably activate mMPO-BBζ or non-transduced (NTD) T cells (**Supplementary Figures 6A, B**). In killing assays with 5D2–2 BCR Nalm6, addition of soluble 5D2–2 IgG reduced mMPO-BBζ–mediated depletion of 5D2–2 BCR target cells in a dose-dependent manner across multiple E:T ratios, consistent with competitive inhibition. In contrast, αhCD19-BBζ cytolysis was unaffected by 5D2–2 IgG (**Supplementary Figures 6C–H**). Thus, occupancy by soluble anti-MPO IgG can impair mMPO CAAR cytolysis *in vitro* without directly triggering off-target activation.

**Figure 5:**
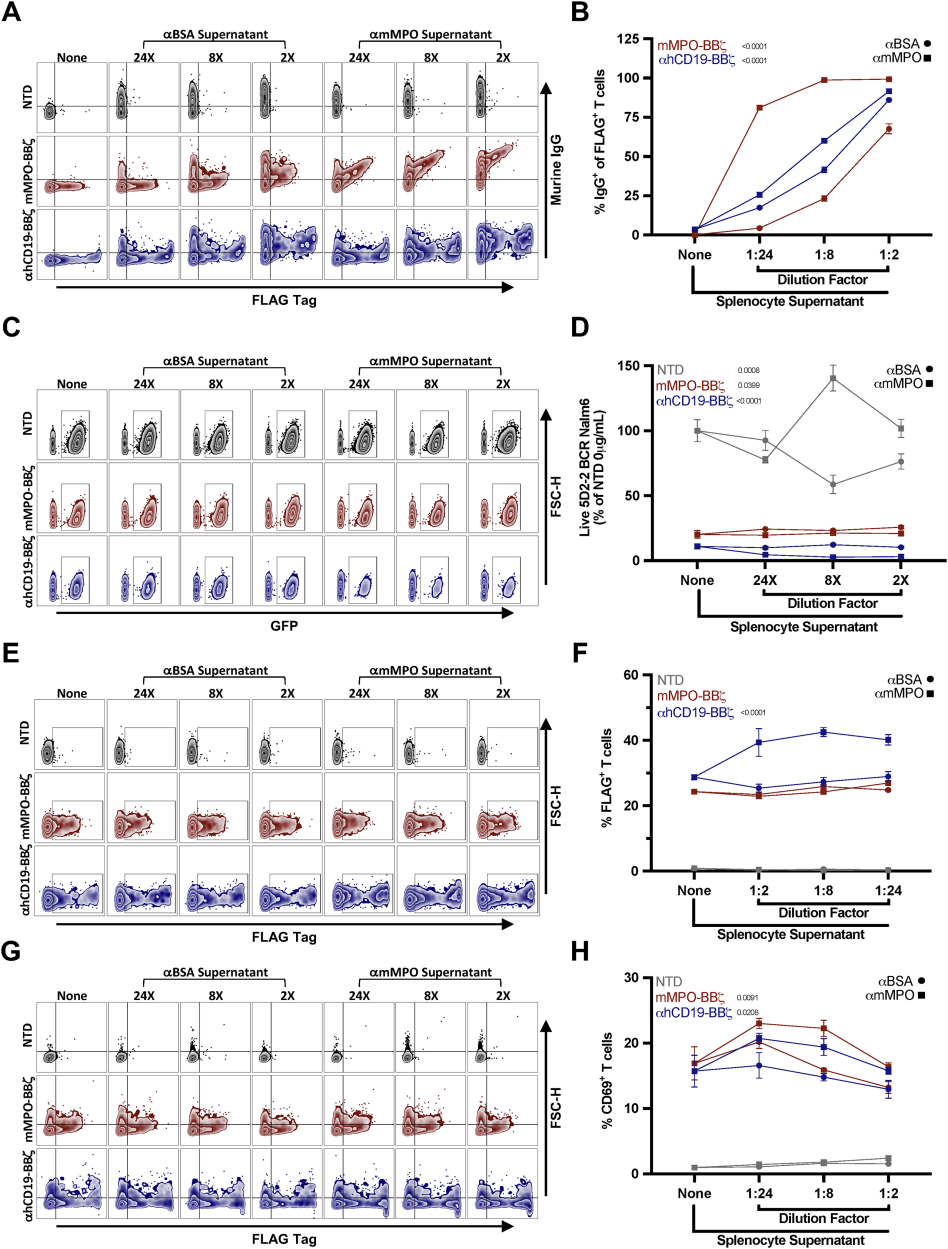
*In-vivo* generated anti-mMPO immunoglobulin binds mMPO-BBζ but does not substantially inhibit cytolysis *in vitro*. **(A)** Representative flow cytometry plots showing murine IgG occupancy (anti-mouse IgG staining) versus CAAR surface expression (anti-FLAG) on T cells following exposure to anti-BSA or anti-mMPO supernatants at the indicated dilutions. **(B)** Quantification of murine IgG occupancy on T cells across supernatant conditions shown in **(A)** graphed as the % of CAAR^+^ T cells bound to IgG. **(C)** Representative flow cytometry plots showing live GFP^+^ 5D2–2 BCR Nalm6 cells after co-culture with NTD, mMPO-BBζ, and αhCD19-BBζ T cells in the absence (“None”) or presence of anti-BSA or anti-mMPO splenocyte culture supernatants (estimated 0.006 μg/ml and 0.1 μg/ml anti-mMPO IgG, respectively) at indicated dilutions (24×, 8×, 2×) **(D)** Quantification of Nalm6 target survival across supernatant conditions shown in **(C)**, normalized to NTD T cells with no supernatant. **(E)** Representative flow cytometry plots showing CAAR surface expression (anti-FLAG) on T cells following exposure to anti-BSA or anti-mMPO supernatants at the indicated dilutions. **(F)** Quantification of CAAR surface expression across supernatant conditions shown in **(E)** graphed as %FLAG^+^ T cells. **(G)** Representative flow cytometry plots showing T cell activation following exposure to anti-BSA or anti-mMPO supernatants at the indicated dilutions. **(H)** Quantification of T cell activation across supernatant conditions shown in **(G)** graphed as %CD69^+^ T cells. Data are shown as mean ± SEM. Statistical significance for **(B, D, F, H)** was assessed for anti-BSA versus anti-mMPO splenocytes within each T cell group by two-way ANOVA.

Since disease-associated anti-MPO IgG potentially neutralizes mMPO-BBζ T cell activity, we tested whether supernatant from splenocytes of MPO^−/−^ mice that were immunized with MPO and expected to have a polyclonal repertoire impacted MPO CAAR function. mMPO-BBζ T cell cytolysis of 5D2–2 Nalm6 targets remained largely unchanged across all supernatant dilutions tested (**Figures 5A, B**). Consistent with CAAR engagement, mMPO-BBζ T cells cultured in supernatants from anti-MPO-immunized cultures increased IgG occupancy on mMPO-BBζ T cells compared with anti-BSA controls (**Figures 5C, D**). Despite this increased binding, CAAR surface expression and T cell activation were not consistently reduced across conditions (**Figures 5E–H**). These findings suggest that, under these *in-vitro* conditions, polyclonal anti-MPO IgG can occupy the CAAR without meaningfully suppressing cytolytic function, contrasting the directly competitive saturating conditions observed with 5D2–2 monoclonal IgG (**Supplementary Figures 6C–S6H**).

## Discussion

ANCA produced by autoreactive B cells are key drivers of pathology in ANCA vasculitis (8, 27, 28). Accordingly, B cell depletion with the anti-CD20 monoclonal antibody rituximab induces remission in the majority of patients; however, more than half ultimately relapse after B cell repopulation, necessitating repeated or continuous treatment to sustain disease control (14, 16, 29, 30). The goal of this study was to evaluate anti-MPO BCR-targeting CAAR T cells as a target-specific and potentially safer alternative to global B cell depletion. Our findings demonstrate that mMPO CAAR T cells efficiently recognize and eliminate anti-mMPO BCR-expressing B cell lines both *in vitro* and *in vivo* while maintaining CAAR expression after antigen engagement. Importantly, mMPO CAAR T cells deplete primary autoreactive murine B cells in a setting that mimics the polyclonal population of pathogenic anti-MPO B cells present in patients (31–33). Together, these findings support the feasibility of selectively targeting MPO-autoreactive B cells with a CAAR T cell approach, enabling durable disease control without broad depletion of the total B cell compartment.

Anti-CD19 CAR T cells were developed as a one-time therapy for B cell malignancies that induce durable remission through prolonged CD19^+^ B cell aplasia, with persistence and/or long-term B cell aplasia reported for more than a decade in some patients (34, 35). We hypothesized that mMPO CAAR T cells could retain key cytotoxicity and persistence advantages of anti-CD19 CAR T cells while sparing non-autoreactive B cells. Across *in-vitro* assays, mMPO CAAR T cells demonstrated effector function against cognate targets comparable to anti-CD19 CAR T cells, with similar activation profiles and antigen-dependent enhancement of surface receptor expression. *In vivo*, mMPO CAAR T cells controlled growth of anti-MPO BCR-expressing Nalm6 and demonstrated similar persistence as anti-CD19 CAR T cells. Interestingly, though MPO produces harmful radicals to aid in the breakdown of pathogens and atypical expression has been linked to cardiovascular disease and cancer, we saw no signs of toxicity in mice who received MPO CAAR T cells (36). It is possible that the CAAR structure impacts the enzymatic function of MPO, reducing the risk of toxicity. Moreover, in primary splenocyte cultures containing autoreactive anti-MPO B cells, both mMPO CAAR T cells and anti-CD19 CAR T cells reduced anti-mMPO IgG titers, supporting functional targeting of the pathogenic B cell response.

The clinical benefits of rituximab and anti-CD19 CAR T cells come at the cost of long-term broad suppression of humoral immunity. Profound B cell depletion or sustained B cell aplasia increases susceptibility to severe infection and sepsis, risks that are particularly consequential in ANCA vasculitis, where infection is a leading cause of early mortality during remission-induced therapy (15, 17). Anti-CD19 CAR T cells are now being evaluated in clinical trials for ANCA vasculitis, and while early case reports have not described severe toxicities, the safety profile in this population remains incompletely defined. Experience from oncology and other autoimmune indications suggests important liabilities with infections common during the immediate post-treatment period and prolonged immunosuppression even after lymphocyte repopulation (37–39). In addition, durable loss of protective humoral responses, including diminished responses to certain vaccinations such as SARS-CoV-2, has been observed following anti-CD19 CAR T cell therapy (24, 40).

Consistent with an antigen-restricted mechanism, mMPO CAAR T cells lysed only targets expressing the cognate anti-MPO BCR *in vitro* and controlled growth of anti-MPO BCR-expressing tumors *in vivo*. In primary splenocyte co-cultures, phenotyping revealed preservation of non-target immune subsets in the broader B cell compartment, in contrast to the near-complete depletion observed with CD19 CAR T cells while retaining depletion of anti-mMPO IgG. Together, these findings suggest that anti-MPO BCR-targeting CAAR T cells could offer a path to durable disease control while mitigating the infectious risk associated with generalized humoral immunodeficiency.

A further advantage of mMPO CAAR T cells can potentially be found in the antibody-producing compartments that are incompletely addressed by current therapies. Anti-CD19 CAR T cells have limited activity against plasma cells in part because CD19 expression becomes heterogeneous and is often downregulated with B cell differentiation (41, 42). Similarly, rituximab does not deplete long-lived plasma cells, which allows for sustained autoantibody production and may contribute to relapse after B cell–directed therapy (43, 44). This interpretation is further supported by the observation that many patients maintain stable autoantibody titers despite achieving clinical remission (30, 45). In contrast, while antibody-secreting cells and memory B cells can exhibit reduced expression of canonical B cell markers, many retain measurable surface BCR. Because mMPO CAAR T cells recognize antigen specificity via the BCR/surface Ig rather than a lineage marker, this strategy could better target MPO-autoreactive memory and antibody-secreting populations than rituximab or anti-CD19 CAR T cells (46), improving durability of autoantibody suppression and reducing relapse risk.

Circulating anti-mMPO immunoglobulin represents a potential barrier to anti-mMPO BCR-targeting CAAR T cell activity *in vivo*, competing for CAAR binding sites and limiting access to BCR-displayed antigen. Similarly, soluble antibody interference has been noted in other CAAR T cell studies (47–49), although cytotoxic functions were not completely eliminated in those settings. In our system, soluble 5D2–2 IgG partially impeded mMPO CAAR T cell killing of Nalm6 cells expressing the 5D2–2 BCR, consistent with direct competition between free immunoglobulin and target BCR for CAAR engagement. In contrast, soluble immunoglobulin produced by primary autoreactive splenocyte cultures did not measurably reduce target control. While the concentration of anti-mMPO IgG was lower in the primary B cell supernatant than the 5D2–2 IgG used, mMPO CAAR T cells exhibited substantial IgG occupancy in these co-cultures. We speculate that this difference reflects the polyclonal nature of the anti-MPO response in primary cells, which comprises antibodies with heterogeneous affinities and epitope specificities, as well as variability in BCR clonotypes on autoreactive B cells, features that reduce the likelihood that any single soluble IgG species will fully block CAAR access across the target population. Notably, this polyclonal model more closely mirrors the autoreactive B cell compartment in patients with ANCA vasculitis (31–33, 50), supporting its relevance for anticipating therapeutic performance *in vivo*.

These studies provide a potentially viable option to selectively target autoreactive B cells and their progeny while sparing the broader B cell pool. Further studies are warranted to test the efficacy of MPO CAAR T cells in a vasculitis model. Although we generated primary autoreactive splenocyte cultures to model a small, polyclonal target population, we did not evaluate therapeutic efficacy in an immunecompetent *in vivo* vasculitis model with quantifiable end-organ injury. Multiple murine models of MPO-ANCA vasculitis have been described, including a passive splenocyte transfer approach that most closely recapitulates disease hallmarks; however, these models depend on intact adaptive immunity (8, 9). Because adoptive T cell administration typically requires lymphodepletion to support engraftment and expansion, implementing this approach in immunecompetent vasculitis models presents a conceptual challenge: autoreactive T cells are important effectors in MPO-ANCA pathogenesis, and lymphodepletion would be expected to blunt or abrogate disease manifestations, complicating interpretation of therapeutic impact (9, 51–53). In addition, the translational feasibility of CAAR T cell therapy is constrained by the cost and complexity of viral vector manufacturing and individualized cell production, limitations shared with CD19 CAR T cell therapies. As a result, early clinical application would most plausibly be directed toward patients with severe, persistently relapsing disease or those with inadequate response or resistance to rituximab-based regimens (22).

Moving toward clinical translation will require validation with human reagents and human disease samples. Key next steps include testing human MPO CAAR T cells against autoreactive B cells derived from patients with MPO-ANCA vasculitis to confirm selective recognition across diverse BCR clonotypes and to assess susceptibility to interference by polyclonal circulating anti-MPO IgG. In parallel, potential off-target binding and toxicity should be evaluated using human tissue panels to assess whether the CAAR construct engages non-B cell targets, helping to define a safety profile suitable for in-human development. While this study focused on MPO-specific ANCA vasculitis, development of CAAR T cells to target PR3 autoreactive B cells should also be evaluated in future studies as a targeted therapy for patients with PR3-specific ANCA vasculitis.

This study highlights the potential for mMPO CAAR T cells to address key limitations of current therapies for MPO-ANCA vasculitis by coupling a well-established cellular therapy platform with antigen-specific targeting of the autoreactive B cell compartment. By selectively eliminating anti-MPO BCR-expressing cells while sparing the broader B cell pool, our approach is designed to retain the efficacy associated with B cell depletion therapies while reducing the infectious risk that accompanies global humoral immunosuppression.

## Materials and methods

### CAAR/CAR T cell generation

To generate the murine MPO CAAR construct (mMPO-BBζ), murine MPO pro-peptide, heavy chain, and light chain coding sequences were codon-optimized, fused to an N-terminal DYKDDDDYK (FLAG tag), synthesized (Integrated DNA Technologies), and cloned into a pHR (54) lentiviral vector with a human CD8α hinge and transmembrane domain followed by 4-1BB and CD3ζ signaling domains under an EF1α promoter. Anti-CD19 CAR ( αhCD19-BBζ or αmCD19-BBζ), 5D2–2 anti-mMPO CAR (5D2-2-BBζ, and 6G1–1 anti-mMPO CAR (6G1-1-BBζ) were generated by replacing the mMPO extracellular domain with an anti-human or anti-mouse CD19 scFv (N-terminal FLAG), 5D2–2 scFv, or 6G1–1 scFv in the same vector backbone.

Primary human T cells were obtained from healthy adult donors through the University of Pennsylvania Human Immunology Core following written informed consent under an IRB-approved protocol (IRB #705906). T cells were cultured in R10 medium (RPMI 1640 supplemented with 10% fetal bovine serum (FBS) (Gibco, Grand Island, NY, #16140-071), 1% HEPES (Gibco, #15630-080), 1% GlutaMAX (Gibco, #35050-061), and 1% penicillin-streptomycin (Gibco, #15140-122) and stimulated for 16h with anti-CD3/CD28 Dynabeads (Thermo Fisher Scientific, #40203D) prior to lentiviral transduction with mMPO-BBζ, αhCD19-BBζ, αmCD19-BBζ 5D2-2-BBζ, or 6G1-1-BBζ. Beads were removed magnetically on day 5, cultures were expanded with 30 IU/ml interleukin 2 (IL-2), and cell density was maintained at 0.75 × 10^6^ cells/ml. On day 7, T cells were used for assays or cryopreserved and subsequently thawed and cultured in R10 and IL-2 24h prior to use.

### CAAR T cell stimulation assays

To evaluate anti-mMPO IgG-specific stimulation of mMPO CAAR T cells, we bound 1 μg/ml, 3 μg/ml, or 10 μg/ml 5D2–2 IgG or an irrelevant IgG (5E5) to high-binding plates overnight in carbonate-bicarbonate buffer. NTD or mMPO-BBζ T cells were cultured for 48h on the plates with bound antibodies after PBS wash. Unstimulated or PMA/Ionomycin-stimulated T cells were used as positive and negative controls, respectively. T cell stimulation was assessed by flow cytometry and IFN-γ Enzyme-linked immunosorbent assay (ELISA).

### Sequencing of 5D2–2 hybridoma anti-mMPO IgG

To sequence the variable heavy and light chains of 5D2–2 and 6G1–1 anti-mMPO IgG, we first isolated mRNA. 5 × 10^6^ cryopreserved 5D2–2 or 6G1–1 anti-mMPO hybridoma cells were thawed and RNA extracted with RNeasy Mini Kit (Qiagen, #74104). cDNA was synthesized from isolated 5D2–2 or 6G1–1 mRNA via the SuperScript III First-Strand Synthesis System (Thermo Fisher Scientific, #18080051) according to the manufacturer’s instructions and with chain-specific RT primers (mIGHG-RT: 5′-AGCTGGGAAGGTGTGCACAC-3′; mIGK-RT: 5′-TTGTCGTTCACTGCCATCAATC-3′). A universal amplification sequence was added to synthesized cDNA with the oligo 5′-AAGCAGTGGTATCAACGCAGAGTACATGRGRGR-3′. The heavy and light (kappa and lambda) variable domains of 5D2–2 and 6G1–1 IgG were PCR amplified with the universal PCR forward primer (ISPCR: 5′-AAGCAGTGGTATCAACGCAGAG-3′) and chain-specific PCR primers (mIGHG-PCR: 5′-GGGATCCAGAGTTCCAGGTC-3′; mIGK-PCR: 5′-ACATTGATGTCTTTGGGGTAGAAG-3′) and gel-extracted, then ligated into a blunt TOPO vector (Invitrogen, #K2800), and transformed into One Shot™ TOP10 chemically competent *E. coli* (Invitrogen, #C404003) and sequenced (Azenta Genewiz, M13F and M13R primers).

### Cell lines

To develop mMPO surface-expressing cell lines, HEK293T cells were transduced with mMPO-BBζ lentivirus and purified by FACS sorting on a FACSAria Fusion Flow Cytometer (BD Biosciences). WT and mMPO-expressing HEK293T were cultured in R10 medium and maintained in logarithmic growth throughout the study.

To develop Nalm6 cell lines, membrane-expressed 5D2-2-TM and 6G1-1-TM were synthesized as 5D2–2 or 6G1–1 IgG light chain, Gly_6_Ser linker, heavy chain, and transmembrane domain (Integrated DNA Technologies) and cloned into a third-generation, self-inactivating lentiviral vector (pTRPE) (55) downstream of an EF1α promoter. 5D2–2 and 6G1–1 BCRs were constructed by inserting the variable heavy and light chains into a BCR structure provided by the Bhoj Lab (University of Pennsylvania) (heavy chain, membrane IgG4, F2A self-cleaving peptide, human growth hormone [HGH] signal peptide, light chain, Ig Kappa, and V5 Tag) and cloned into the pHR lentiviral vector. Nalm6 cells expressing Click Bettle Green (CBG) luciferase and GFP (CBG-GFP^+^ Nalm6) were transduced with 5D2-2-TM, 6G1-1-TM, 5D2–2 BCR, or 6G1–1 BCR lentivirus and FACS-sorted on FACSAria Fusion Flow Cytometer. WT, 5D2-2-TM, 6G1-1-TM, 5D2–2 BCR, and 6G1–1 BCR CBG-GFP^+^ Nalm6 were cultured in R10 medium and maintained in logarithmic growth throughout the study.

### Impedance-based cytotoxicity assay

WT and mMPO-expressing HEK293T cell lines (2 × 10^4^) were seeded in E-plate VIEW plates (Agilent, #300 601 020) and allowed to adhere for 24h prior to addition of T cells. NTD, 5D2-2-BBζ, or 6G1-1-BBζ effector cells were added at 3:1 and 1:1 effector to target (E:T) ratios. HEK293T cells cultured without effectors or with 10% SDS were used as spontaneous and total lysis controls, respectively. Impedance (cell index) measurements were collected every 15 min for 120h on an xCELLigence RTCA eSight System (Agilent). Percent specific lysis was calculated at the time of peak target cell index, normalized for each target cell line so that the average of the total lysis controls was 0, and calculated as 100 × (spontaneous lysis – X)/spontaneous lysis).

### Luciferase-based cytotoxicity assay

WT, 5D2-2-TM, and 6G1-1-TM expressing CBG-GFP^+^Nalm6 cell lines (2 x 10^4^) were co-cultured with NTD or mMPO-BBζ effector cells at 3:1 and 1:1 E:T ratios. Nalm6 cultured without effectors and with 10% SDS was used as a spontaneous and total lysis control, respectively. After 48h of co-culture, cell survival was measured by quantification of intracellular CBG luciferase. First, cells were washed and lysed with Reporter Lysis 5× Buffer (Promega, #E1501) and frozen at −80°C. Plates were thawed and 40 μl of supernatant combined with 50 μl of luciferase substrate (Promega, #E1501). Luciferase activity was measured as luminescence on a Synergy H4 Hybrid Plate Reader. Percent specific lysis was calculated by first setting the average of total lysis controls to zero, then calculated as 100 × (spontaneous lysis – X)/spontaneous lysis).

### Flow-cytometry-based killing assay

WT, 5D2–2 BCR, and 6G1–1 BCR CBG-GFP^+^Nalm6 (1 × 10^5^) were co-cultured at 1 × 10^6^ cells/ml with NTD, mMPO-BBζ, or αhCD19-BBζ effector cells at 1:1 or 1:3 E:T ratios. After 48h of co-culture, live Nalm6 cells were counted by flow cytometry and normalized to Precision Count Beads (BioLegend, #424902) (live Nalm6/(beads/500). Nalm6 cell survival was calculated as the fold change from the average of live Nalm6 cells co-cultured with NTD T cells. For repeated stimulation assays, half of the media was replaced, and WT, 5D2–2 BCR, and 6G1–1 BCR CBG-GFP Nalm6 were added to existing co-cultures at 1 × 10^6^ cells/ml every two days for three stimulations.

### Murine tumor model

All experiments were performed via protocols approved by the Institutional Animal Care and Use Committee of the University of Pennsylvania. Nod *scid* gamma (NSG; Nod.Cg-Prkdc^scid^Il2rg^tm1Wjl/SzJ^) mice were obtained from the University of Pennsylvania Stem Cell and Xenograft Core and housed under specific pathogen-free conditions. On day -1, 5D2–2 BCR CBG-GFP Nalm6 (1 × 10^6^) was injected *I.V.* into male and female NSG mice. The next day, baseline bioluminescence imaging (BLI) was performed using an IVIS Lumina S3 (PerkinElmer) 8 min after *I.P.* injection of 100 μl D-luciferin (Gold Biotechnology, #LUCK-100, 30 mg/ml) under isoflurane anesthesia. Mice were randomized by sorting baseline luminescence from low to high and then assigned in ascending order to groups. Mice were treated *I.V.* with 2 × 10^6^ mMPO-BBζ CAAR^+^ T cells (*n* = 7) or 2 × 10^6^ αhCD19-BBζ CAR^+^ T cells (*n* = 10). Mice were treated with NTD T cells equivalent to the total number of T cells in the mMPO-BBζ group (*n* = 10), and untreated mice (*n* = 6) served as negative controls. Tumor burden was monitored by BLI twice weekly and expressed as both total flux (photons/second) and as fold change from 9 days post-T cell treatment. Weight change was monitored twice weekly and expressed as fold change from baseline (day 0). Blood was sampled retro-orbitally on day 28 to assess T cell persistence.

### Generation and culture of primary autoreactive murine B cells and total splenocytes

All experiments were performed via protocols approved by the Institutional Animal Care and Use Committee of the University of Pennsylvania. MPO^−/−^ mice were obtained from Jackson Laboratories (Stock #004265) and maintained with homozygous breeding pairs. Male and female mice at least 8 weeks old were used in experiments. MPO^−/−^ mice were immunized with 10 μg mMPO in Complete Freund’s Adjuvant (Sigma-Aldrich, #F5881) *I.P.* on day 0, then 10 μg mMPO booster injections in Incomplete Freund’s Adjuvant (Sigma-Aldrich, #F5506) on days 21 and 35. Three days prior to splenocyte harvest—no less than 2 weeks after day 35—mice were given a final *I.P.* injection of 10 μg mMPO in PBS. MPO^−/−^ mice were immunized with BSA on the same schedule as negative controls. Blood was collected from the tail vein once per week to monitor the development of the serum anti-mMPO IgG titer by ELISA.

For B cell culture, immunized MPO^−/−^ mice were sacrificed, blood was collected via cardiac puncture, and the spleens were harvested. Spleens were crushed and splenocytes filtered through a 100 μM filter (Falcon, #352360) in MACS buffer (0.5% BSA in autoMACS rinsing solution (Miltenyl Biotec, #130-091-222)). Pan B cells were isolated with an EasySep Mouse Pan-B cell Isolation Kit (StemCell Technologies, #19844) per manufacturer’s instructions. Isolated B cells were cultured at 1 × 10^6^ cells/ml in B cell media (RPMI 1640, 10% FBS, 1% GlutaMax, 1% Pen/Strep, 1% Non-essential Amino Acids (Gibco, #11140-050), 1 mM 2-mercaptoethanol) in plasma cell-differentiating conditions with 10 ng/ml mIL-4 (Peprotech, #214-14), 5 ng/ml mIL-5 (Peprotech #215-15), 5 μg/ml lipopolysaccharide (Sigma-Aldrich, #L3012) for three days.

For splenocyte culture, immunized MPO^−/−^ splenocytes were harvested as above and pooled (*n* = 11 mMPO-immunized and *n* = 4 BSA-immunized mice). Red blood cells were lysed with ACK buffer (Quality Biological, #118-156-101) for 2 min. Splenocytes were cultured at 2 × 10^6^ cells/ml in B cell media under plasma cell differentiating conditions for three days.

### Primary autoreactive B cell depletion assays

Primary B cells (4 × 10^6^) from mMPO- or BSA-immunized MPO^-/-^ mice were cultured alone or with 4 × 10^6^ NTD- or mMPO-BBζ T cells in B cell media under plasma cell differentiating conditions for 3 days. Primary splenocytes from BSA- or mMPO-immunized MPO^-/-^ mice were also pooled by immunization and cultured alone or with mMPO-BBζ or αmCD19-BBζ T cells at 3 × 10^6^ CAAR^+^/CAR^+^ T cells per mouse or an equivalent number of NTD T cells for three days under plasma cell differentiating conditions. Culture supernatant was collected for ELISA, and cells were stained for analysis by flow cytometry.

### Enzyme-linked immunosorbent assays 

IFN-γ production by T cells was quantified by sandwich ELISA. Cell-free supernatants were harvested after 48h of T cell culture with plate-bound or soluble 5D2–2 anti-mMPO IgG, or WT, 5D2-2-TM, or 5D2–2 BCR Nalm6. T cells alone or stimulated with 25 ng/ml phorbol-12-myristate-13-acetate (PMA) and 1 μg/ml ionomycin were used as negative and positive stimulation controls, respectively. Supernatant IFN-γ concentrations were measured using the Human IFN-γ DuoSet ELISA Kit (R&D Systems, #DY285B) per manufacturer’s instructions.

Anti-mMPO IgG was quantified by sandwich ELISA. 0.5 μg/ml mMPO, purified in-house or commercial recombinant (R&D Systems, #3667-MP), was coated on high-binding ELISA plates (Revity, #6055620) overnight at room temperature. Plates were washed with 0.05% Tween-20 and blocked with 1% BSA (Sigma-Aldrich, #A7906) for 2h at room temperature. After washing, MPO^-/-^ serum or B cell/splenocyte culture supernatant (cell-free) was added to the plate overnight at 4°C. Anti-mMPO IgG was detected with anti-mouse IgG HRP-linked antibody (Cell Signaling Technology, #7076S) for 1h at room temperature. ELISA was read with 100 μl TMB (Cell Signaling Technology, #7004P6) for 20 min, followed by 50 μl stop solution (2N H_2_SO_4_). Absorbance at 450 nm was measured on a Synergy H4 Hybrid Plate Reader with 540 nm reference.

### Western blot

9.75 μl of WT or 8xHis-mMPO-secreting HEK293T supernatant or 15 μg of purified 8xHis-mMPO or commercial recombinant mMPO were electrophoresed on a NuPAGE 4%–12% Bis-Tris Gel (Invitrogen, #NP0336) and transferred onto an Odyssey Nitrocellulose membrane (LICORbio, #926-31090). Revert 700 Total Protein Stain (LiCORbio, #926-11010) was performed according to the manufacturer’s instructions and imaged on the LiCOR Odyssey Imaging System. Following stain removal, the membrane was blocked for 1h in Intercept Blocking Buffer (LiCORbio, #927-60001). Membranes were incubated overnight in 5D2–2 anti-mMPO IgG in Intercept Blocking Buffer. IRDye 800CW goat anti-mouse IgG (LiCORbio, #926-32232) was added to membranes for 1h in Intercept Blocking Buffer at room temperature. Membranes were imaged on LiCOR Odyssey Imaging System.

### 8xHis mMPO production and FPLC purification

The 8xHis-mMPO secreting construct was synthesized as murine MPO with IL-2 signal peptide, 8x His tag, mMPO heavy and light chains, a P2A self-cleaving peptide, and a puromycin resistance gene and cloned into the pTRPE lentiviral vector. HEK293T cells were transduced with 8xHis-mMPO lentivirus and positively selected in 5 ug/ml puromycin (Thermo Fisher Scientific, #AAJ67236XF). Supernatant from 8xHis-mMPO culture was purified by fast protein liquid chromatography (FPLC). Briefly, a HisPur Ni-NTA Chromatography Cartridge (Thermo Fisher Scientific, #90098) was equilibrated with equilibration buffer (20 mM sodium phosphate and 500 mM sodium chloride). Supernatant was diluted 1:1 with equilibration buffer containing 60 mM imidazole and loaded onto the column. The column was washed with 20 mM sodium phosphate, 500 mM sodium chloride, and 50 mM imidazole and eluted using a gradient of 50–200 mM imidazole in equilibration buffer. Eluted 8xHis-mMPO was dialyzed in PBS using Amicon Ultra 30K centrifugal filters (Millipore, #UFC903008).

### Flow cytometry

Viability of all cells was assessed with a fixable viability dye (Live/Dead Violet; Life Technologies, #L34964A). Surface mMPO CAAR and CD19 CAR expression were detected with PE-conjugated anti-DYKDDDDYK (FLAG) antibody (PE; BioLegend, #637310). Surface 5D2–2 scFv expression was detected with biotin-SP-conjugated F(ab’)2 anti-mouse IgG antibody (Jackson ImmunoResearch, #115-065-072) followed by streptavidin-PE (BD Biosciences, #554061). Surface BCR expression was detected with anti-V5 Tag (PE; Thermo Fisher Scientific, 12-6796-42). Human T cells were identified as CD3^+^ (BV605, BD Biosciences, #563217) and further gated with anti-human CD4 (BUV395; BD Biosciences, #563550) and anti-human CD8 (BUV805, BD Biosciences, #612889). For *in vivo* persistence, peripheral blood was collected into EDTA tubes and enumerated using BD Trucount tubes (BD Biosciences, #340334). T cell activation was assessed by CD69 staining using anti-human CD69 (BV711; (BioLegend, #310944) or anti-human CD69 (FITC; BioLegend, #310904). Cell populations in primary murine splenocytes were characterized using a 12-color antibody panel (**Supplementary Table 1**) and visualized by t-SNE following OR-gating on lineage-positive events and down-sampling; major populations included human T cells, murine T cells, B cells, plasmablasts/plasma cells, NK cells, and neutrophils. All flow cytometry data was analyzed with FlowJo10.

### Statistics

GraphPad Prism 10 was used for all analyses. Groups are graphed as mean ± SEM. Group means were compared with *t* tests with Welch’s correction, or one-way/two-way ANOVA with Tukey’s multiple comparisons test or uncorrected Fisher’s LSD. Mouse survival was analyzed with Kaplan–Meier with log-rank (Mantel Cox) testing. *p* < 0.05 was considered significant.

## Data Availability

The raw data supporting the conclusions of this article will be made available by the authors, without undue reservation.
