# Supplementary material for: Chimeric autoantibody receptor T cells for targeted depletion of myeloperoxidase-specific anti-neutrophil cytoplasmic antibody-producing B cells
Source: Front Immunol. 2026 May 8;17:1814452. doi: 10.3389/fimmu.2026.1814452 (PMC13194496; doi:10.3389/fimmu.2026.1814452)
Supplement: Supplementary file 1 [file DataSheet1.pdf]

Table S1. Flow cytometry panel for identifying primary murine splenocytes

Target	Fluorophore	Company	Catalog #	Cell Population(s)
Mouse anti-human CD3	BV605	BD Biosciences	563217	Human T cell
Hamster anti-mouse CD3e	BUV395	BD Biosciences	563565	Mouse T cell
Anti-mouse CD19	BV650	BioLegend	152427	Mouse B cell/Plasmablast
Anti-mouse/human CD45R/B220	BV510	BioLegend	103248	Mouse B cell/Plasmablast/Plasma cell
Anti-mouse IgD	APC/Cy7	BioLegend	4057115	Mouse B cell
Anti-mouse IgM	PE Dazzle	BioLegend	406530	Mouse B cell
Anti-mouse CD267/TACI	PE	BioLegend	133404	Mouse B cell/Plasmablast/Plasma cell
Anti-mouse CD138 (syndecan-1)	BV605	BioLegend	142540	Mouse Plasmablast/Plasma cell
Rat anti-CD11b	APC	BD Biosciences	561690	Early Mouse B cell/Neutrophil
Anti-mouse Ly-6G/Ly-6C (Gr-1)	FITC	BD Biosciences	108406	Neutrophil
Anti-mouse NK1.1	PE/Cy7	BioLegend	108714	NK Cells

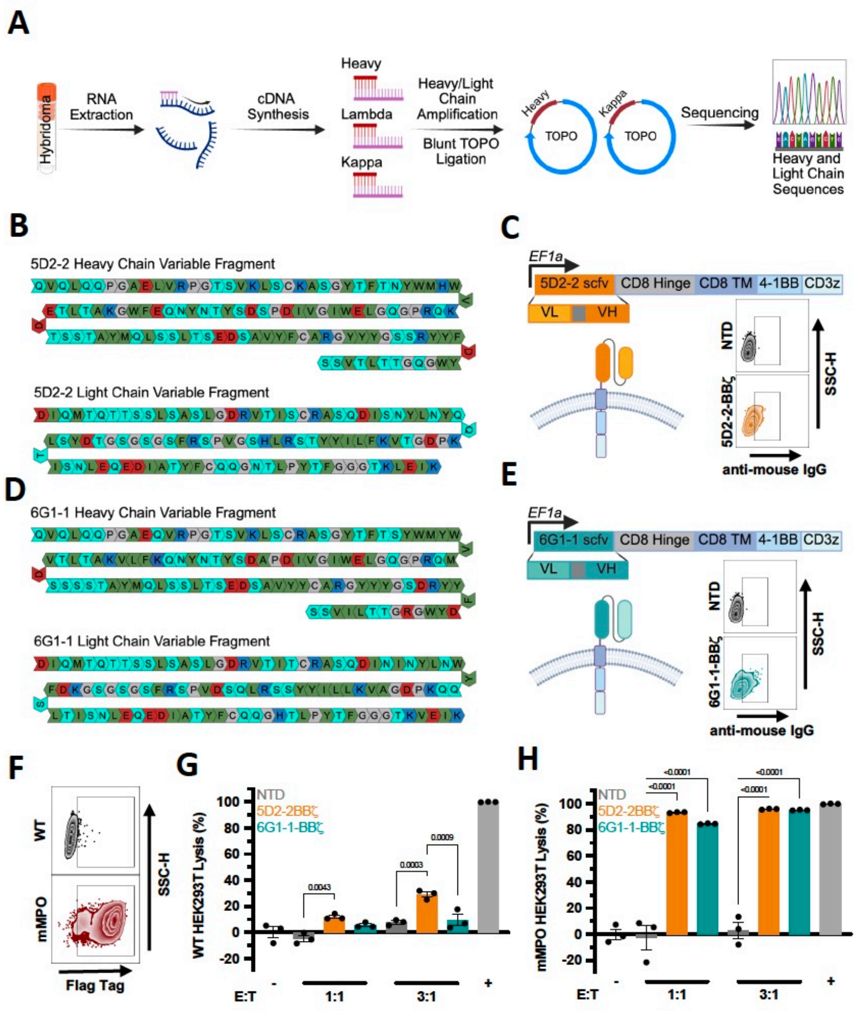

Figure S1. 5D2-2 and 6G1-1 anti-mMPO hybridoma sequencing and scFv development.

(**A**) Workflow for sequencing the 5D2-2 IgG and 6G1-1 IgG heavy- and light-chain variable regions from hybridoma RNA: cDNA synthesis, PCR amplification of Ig variable fragments, cloning into TOPO vector, and Sanger sequencing. (**B**) Amino acid sequences of the 5D2-2 variable heavy and light chains, color-coded by residue class (*light blue – polar uncharged, red – negatively charged, dark blue – positively charged, green – hydrophobic, grey – other*). (**C**) Schematic of the 5D2-2-BBζ CAR T cell construct [VL-(G4S)3-VH single chain variable fragment (scFv); CD8a hinge/transmembrane; 4-1BB;CD3ζ] and flow cytometry of 5D2-2-

BBζ expression in primary human T cells (αmouse Ig staining). (**D**) Amino acid sequences of the 6G1-1 variable heavy and light chains, color-coded by residue class (*light blue – polar uncharged, red – negatively charged, dark blue – positively charged, green – hydrophobic, grey – other*). (**E**) Schematic of the 6G1-1-BBζ CAR T cell construct [VL-(G4S)3-VH single chain variable fragment (scFv); CD8a hinge/transmembrane; 4-1BB;CD3ζ] and flow cytometry of 6G1-1-BBζ expression in primary human T cells (αmouse Ig staining). (**F**) Flow cytometry of mMPO (FLAG) surface expression in WT vs mMPO-transduced HEK293T cells. (**G**) Impedance-based cytotoxicity of NTD, 5D2-2-BBζ, and 6G1-1-BBζ T cells against WT HEK293T targets, normalized to untreated targets. (**F**) Impedance-based cytotoxicity of NTD 5D2-2-BBζ, and 6G1-1-BBζ T cells against mMPO-expressing HEK293T targets, normalized to untreated targets. Data in G and H are shown as mean ± SEM. Statistical significance for E and F was assessed by ordinary one-way ANOVA with Tukey's multiple comparisons test.

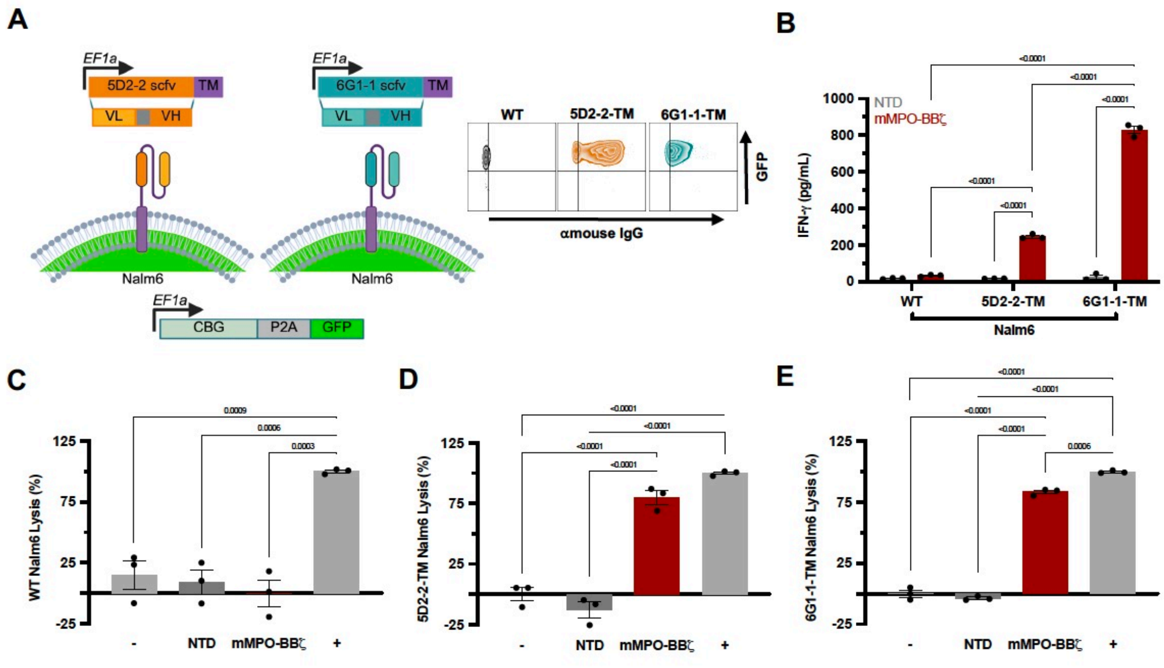

Figure S2. mMPO-BBζ T cells lyse 5D2-2-TM and 6G1-1-TM Nalm6 targets *in vitro*.

(**A**) Schematic of 5D2-2-TM and 6G1-1-TM constructs (5D2-2 scFv fused to a transmembrane domain) and the Click Beetle Green (CBG) luciferase-P2A-GFP reporter cassette (*left*); flow cytometry confirming 5D2-2-TM and 6G1-1-TM expression (αmouse Ig staining) and GFP in Nalm6 cells (*right*). (**B**) IFN-γ secretion by NTD and mMPO-BBζ T cells after 48 h co-culture with WT, 5D2-2-TM, or 6G1-1-TM Nalm6 at 1:1 and 3:1 E:T, measured by ELISA. (**C**) Cytotoxicity of NTD and mMPO-BBζ T cells against WT Nalm6 measured by luciferase signal, normalized to untreated WT Nalm6 and graphed as %maximal lysis (10% SDS). (**D**) Cytotoxicity of NTD and mMPO-BBζ T cells against 5D2-2-TM Nalm6 measured and normalized as in (C). (**E**) Cytotoxicity of NTD and mMPO-BBζ T cells against 6G1-1 measured and normalized as in (C). Data are shown as mean ± SEM. Statistical significance for B was assessed by two-way ANOVA with Sidak's multiple comparisons test. Statistical significance

for C, D, and E was assessed by ordinary one-way ANOVA with Tukey's multiple comparisons test.

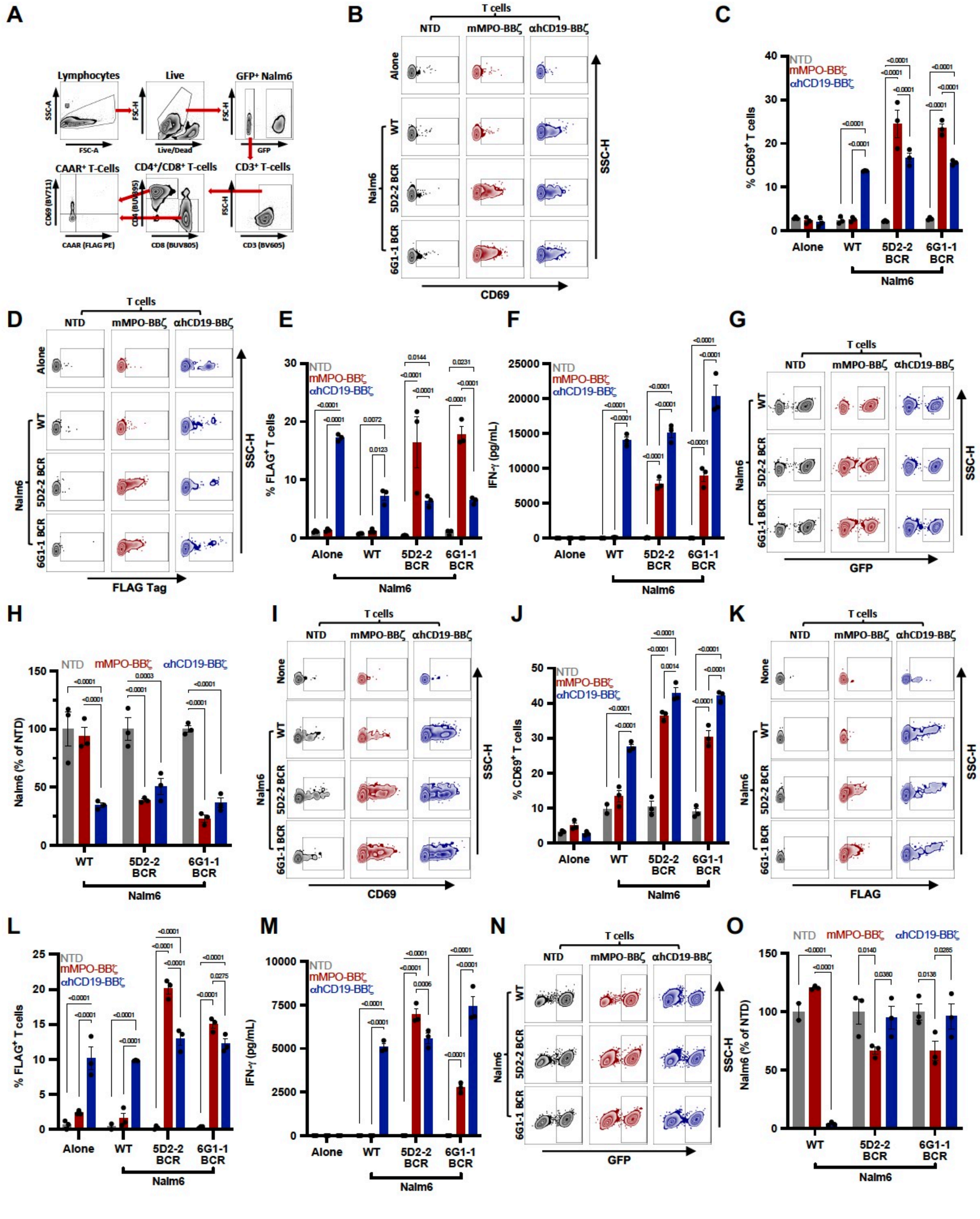

Figure S3. mMPO-BBζ T cells lyse 5D2-2 BCR and 6G1-1 BCR Nalm6 targets at 1:3 E:T *in vitro*

(**A**) Flow cytometry gating strategy for Nalm6 targets and T cell populations after co-culture: live lymphocytes; Nalm6 defined as live GFP^{+}; T cells defined as $GFP^{-}CD3^{+}CD4^{+}/CD8^{+}$; $CAAR^{+}$ defined by FLAG; activation defined by $CD69^{+}$. (**B**) Representative flow cytometry plots showing CD69 expression on NTD, mMPO-BBζ, and αhCD19-BBζ T cells after co-culture. (**C)** Quantification of CD69 expression (% $CD69^{+}$) on T cells alone and after co-culture with WT, 5D2-2 BCR, and 6G1-1 BCR Nalm6. (**D**) Representative flow cytometry plots showing CAAR (FLAG) expression on T cells after co-culture. (**E**) Quantification of CAAR expression (% $FLAG^{+}$) on NTD and mMPO-BBζ T cells alone and after co-culture. (**F**) IFN-γ secretion by NTD, mMPO-BBζ, and αhCD19-BBζ T cells after 48h 1:3 co-culture with WT, 5D2-2 BCR, and 6G1-1 BCR Nalm6, measured by ELISA. (**G**) Representative flow cytometry plots of live Nalm6 (GFP^{+}) after co-culture. (**H**) Quantification of live GFP^{+} Nalm6 targets normalized to counting beads and displayed as fold change relative to NTD. (**I**) Representative flow cytometry plots showing CD69 expression after repeated stimulation. (**J**) Quantification of CD69 expression (% $CD69^{+}$) after repeated stimulation with WT, 5D2-2 BCR, and 6G1-1 BCR Nalm6. (**K**) Representative flow cytometry plots showing CAAR expression after repeated stimulation. (**L**) Quantification of CAAR expression (% $FLAG^{+}$) after repeated stimulation. (**M**) IFN-γ secretion after three consecutive 48h stimulations with WT, 5D2-2 BCR, and 6G1-1 BCR Nalm6 (fresh targets added each round). (**N**) Representative flow cytometry plots showing live GFP^{+} Nalm6 after the third stimulation. (**O**) Quantification of Nalm6 target depletion after repeated stimulation, displayed as fold change relative to NTD. Data are shown as mean ± SEM. Statistical significance for C, E, F, H, J, L, M, and O was

assessed by two-way ANOVA with Sidak's multiple comparisons test (C, F, J, J, M, O) or uncorrected Fisher's LSD (E, L).

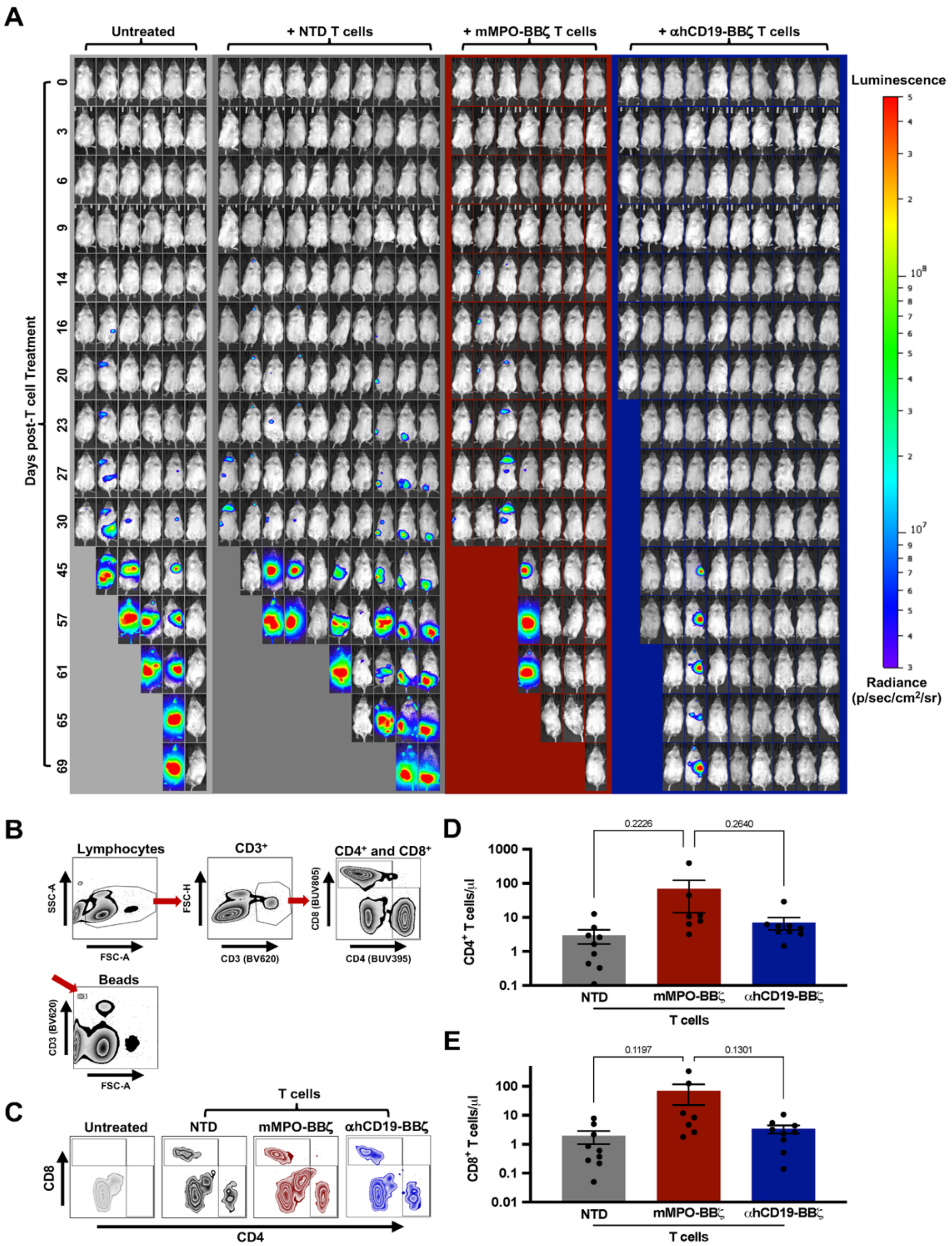

Figure S4. Expanded *in vivo* tumor imaging and gating strategy for peripheral T cell persistence.

(**A**) Complete set of bioluminescence images for individual mice bearing 5D2-2 BCR Nalm6 tumors that were untreated (*light grey*) or treated with NTD (*dark grey*), mMPO-BBζ (*red*), or

αhCD19-BBζ (*blue*) T cells. (**B**) Flow cytometry gating strategy for quantification of peripheral human T cells in NSG mice; events were normalized to counting beads to calculate T cells/μL blood. (**C**) Representative flow cytometry plots of peripheral human $CD3^{+}$ $CD4^{+}$/$CD8^{+}$ T cells in untreated and T cell-treated groups. (**D**) Peripheral human $CD4^{+}$ T cells/μL blood at day 28 post-treatment. (**E**) Peripheral human $CD8^{+}$ T cells/μL blood at day 28 post-treatment. Data in D-E are shown as mean ± SEM. Statistical significance for D-E was assessed by ordinary one-way ANOVA with Tukey's multiple comparisons test.

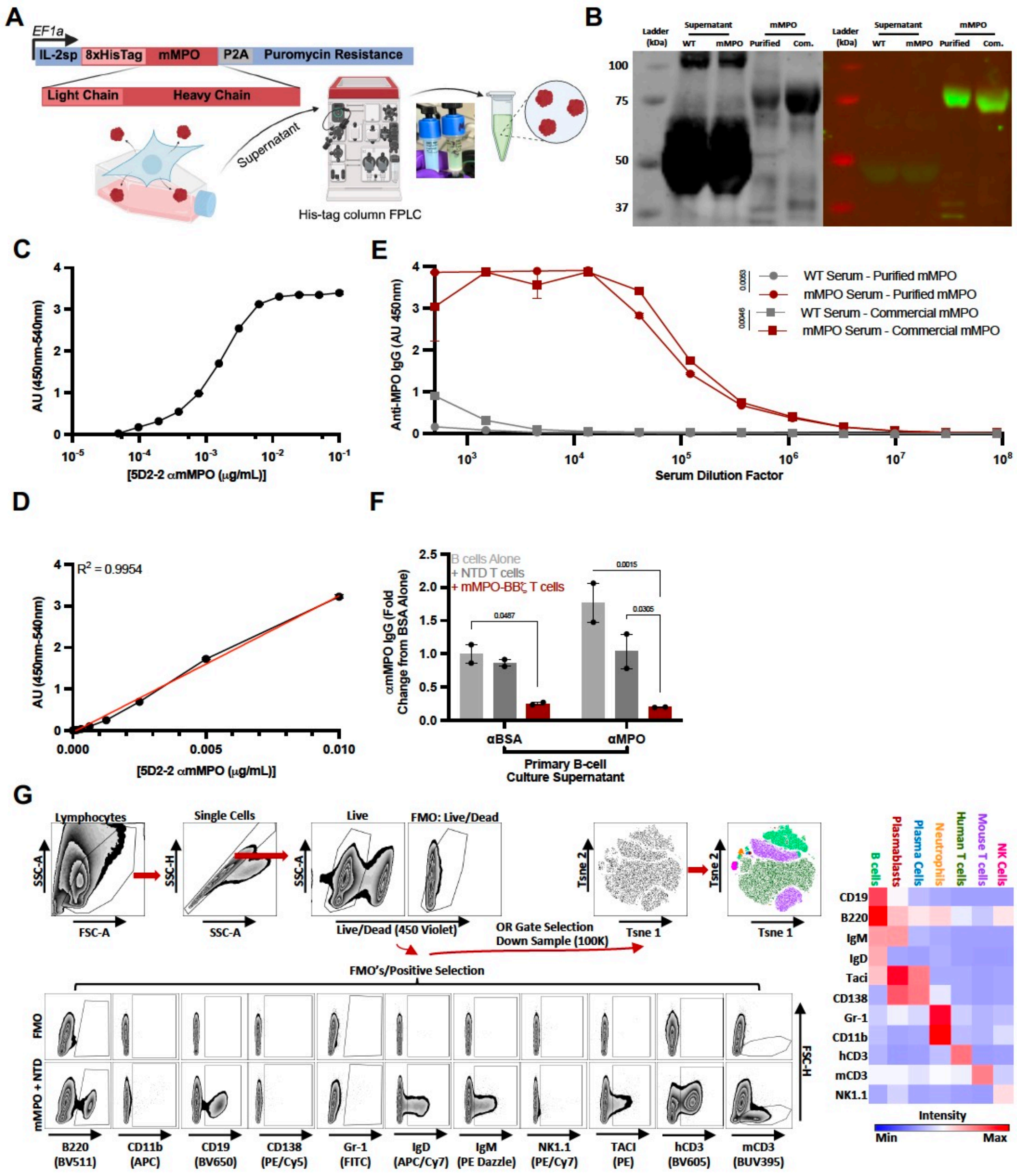

Figure S5. Supporting analyses for mMPO production and primary autoreactive assays

(**A**) Schematic of secreted mMPO production and purification: lentiviral construct encoding IL-2 signal peptide-8x His Tag-mMPO-P2A-puromycin resistance gene; purification of 8xHis-mMPO from transduced HEK293T supernatants by FPLC using a His-tag affinity column. (**B**)

Western blot assessing mMPO purity and identity in unpurified supernatant, purified mMPO, WT HEK293T supernatant, and a commercial recombinant mMPO control; total protein stain and anti-mMPO immunoblot shown. (**C**) Full range 5D2-2 anti-mMPO IgG ELISA standard curve. (**D**) Linear range 5D2-2 anti-mMPO IgG ELISA standard curve used for quantification. (**E**) ELISA comparing anti-mMPO IgG detected using purified 8xHis-mMPO versus commercial recombinant mMPO as coating antigen. (**F**) Anti-mMPO IgG production by pan B-cells from anti-BSA vs anti-mMPO-immunized mice cultured for 3 days alone or with NTD or mMPO-BBζ T cells, quantified by ELISA and displayed as fold change relative to anti-BSA B-cells cultured alone. (**G**) Flow cytometry gating strategy for splenocyte subset identification after culture (live single-cell lymphocytes; marker panel as indicated; FMO controls used for gate setting), including t-SNE visualization and heat map of marker intensity for annotated populations. Data in C-F are shown as mean ± SEM. Goodness of fit in D was calculated with simple linear regression. Statistical significance for E was assessed as paired t-tests. Statistical significance for F was assessed as two-way ANOVA with Tukeys multiple comparisons Panels A, B and G are schematic/representative.

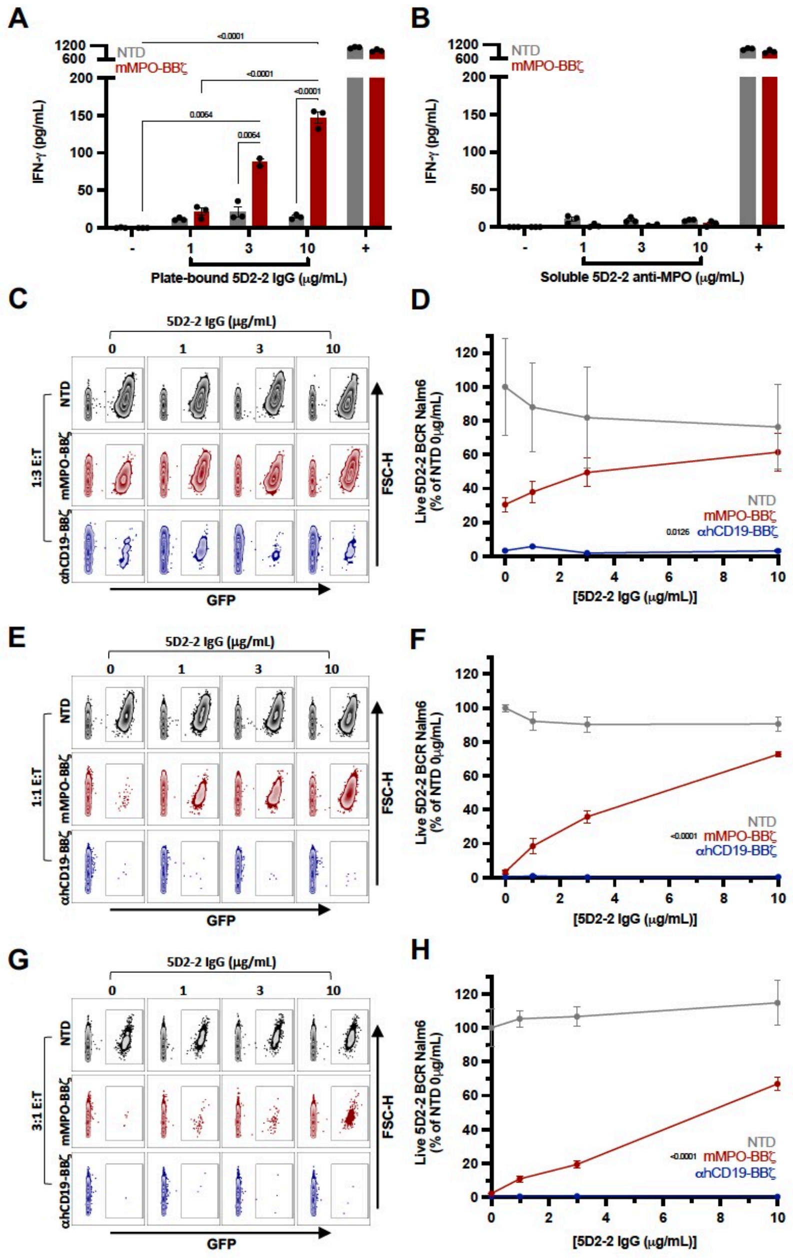

Figure S6. Soluble 5D2-2 anti-mMPO IgG competitively inhibit cytolysis without activating mMPO-BBζ T cells.

(**A**) IFN-γ secretion by NTD and mMPO-BBζ T cells after 48 hour stimulation with plate-bound 5D2-2 IgG at indicated concentrations. Unstimulated and PMA/ionomycin-stimulated T cells serve as negative and positive controls, respectively. (**B**) IFN-γ secretion after 48 hour

incubation of NTD and mMPO-BBζ T cells with soluble 5D2-2 IgG at 1, 3, and 10 μg/mL. Unstimulated (-) and PMA/ionomycin-stimulated (+) T cells serve as negative and positive controls, respectively. (**C**) Representative flow cytometry plots showing live GFP^+ 5D2-2 BCR Nalm6 target cells after 48 hour co-culture with NTD, mMPO-BBζ, and αhCD19-BBζ T cells at 1:3 E:T in the presence of increasing concentrations of soluble 5D2-2 IgG. (**D**) Quantification of 5D2-2 BCR Nalm6 survival for the 1:3 E:T condition shown in (C) across soluble 5D2-2 IgG concentrations, normalized to NTD T cells cultured with no 5D2-2 IgG. (**E**) Representative flow cytometry plots showing live GFP^+ 5D2-2 BCR Nalm6 target cells after 48 hour co-culture with NTD, mMPO-BBζ, and αhCD19-BBζ T cells at 1:1 E:T in the presence of increasing concentrations of soluble 5D2-2 IgG. (**F**) Quantification of 5D2-2 BCR Nalm6 survival for the 1:1 E:T condition shown in (E) across soluble 5D2-2 IgG concentrations, normalized to NTD T cells cultured with no 5D2-2 IgG. (**G**) Representative flow cytometry plots showing live GFP^+ 5D2-2 BCR Nalm6 target cells after 48 hour co-culture with NTD, mMPO-BBζ, and αhCD19-BBζ T cells at 3:1 E:T in the presence of increasing concentrations of soluble 5D2-2 IgG. (**H**) Quantification of 5D2-2 BCR Nalm6 survival for the 3:1 E:T condition shown in (G) across soluble 5D2-2 IgG concentrations, normalized to NTD T cells cultured with no 5D2-2 IgG. Data are shown as mean ± SEM. Statistical significance for panels A-B was assessed by two-way ANOVA with Sidak's multiple comparisons test. Statistical significance of D, F, and H was assessed within each T cell group by ordinary one-way ANOVA.
